# Association between ABO blood groups and SARS-CoV-2 infection in blood donors of Puglia region

**DOI:** 10.1007/s00277-023-05331-1

**Published:** 2023-07-13

**Authors:** Alessia Sticchi Damiani, Antonella Zizza, Federico Banchelli, Maddalena Gigante, Maria Lucia De Feo, Angelo Ostuni, Valerio Marinelli, Serena Quagnano, Pierpaolo Negro, Nicola Di Renzo, Marcello Guido, The Seroprevalence COVID Blood Donors Study Group

**Affiliations:** 1 Immunohaematology and Transfusion Medicine Unit, Inter-Company Department of Transfusion Medicine (IDTM) of Local Health Unit (LHU) of Lecce, Vito Fazzi Hospital, 73100 Lecce, Italy; 2 Institute of Clinical Physiology, National Research Council, Via Prov.Le Lecce-Monteroni, 73100 Lecce, Italy; 3 Department of Medical and Surgical Sciences, University of Modena and Reggio Emilia, Modena, Italy; 4 Unit of Statistical and Methodological Support to Clinical Research, University Hospital of Modena, Modena, Italy; 5 Immunohaematology and Transfusion Medicine Unit, Inter-Company Department of Transfusion Medicine (IDTM) of Local Health Unit (LHU) of Bari, S. Paolo Hospital, Bari, Italy; 6 Immunohaematology and Transfusion Medicine Unit, Inter-Company Department of Transfusion Medicine (IDTM) of Local Health Unit (LHU) of Foggia, Ospedali Riuniti of Foggia, Foggia, Italy; 7 Immunohaematology and Transfusion Medicine Unit, Policlinico of Bari, Bari, Italy; 8 Laboratory of Hygiene, Department of Biological and Environmental Sciences and Technologies, University of Salento, Lecce, Italy

**Keywords:** ABO blood type, COVID-19, Blood donors, SARS-CoV-2 infection

## Abstract

This is an observational multicentric cross-sectional study aiming at assessing the association between ABO blood groups and SARS-CoV-2 seroprevalence among the blood donors in Puglia region. Data on ABO and Rh blood groups and demographic characteristics were obtained from Blood Bank Information System. All donors were screened for SARS-CoV-2 IgG antibodies. Comparison of seroprevalence among blood groups and the association between the recorded variables and seroprevalence were evaluated. A total of 35,709 donors from 22 centers were included, with a seroprevalence of 6.8%. The distribution of ABO phenotypes was blood type O (46.8%), A (34.0%), B (14.7%), and AB (4.5%). Among the 2416 donors reactive for SARS-CoV-2 IgG, the prevalent phenotype was blood type O (43.1%), followed by A (37.7%), B (14.2%), and AB (5%). The seroprevalence of phenotype A and AB was 7.5%, followed by B (6.5%) and O (6.2%). According to the adjusted analysis, there was an increase in seroprevalence in groups A and AB, compared to group O, and an increase in males compared to females. A possible effect modification was observed after stratifying for sex (*p* = 0.0515). A significantly lower prevalence of blood type O was found compared to A and AB, whereas no association was observed between Rh factor and seroprevalence. We hypothesized that the A antigen present in blood type A and AB can play a role in the binding of SARS-CoV-2 to ACE2 receptors, resulting in an increased risk of infection. Furthermore, natural anti-A/anti-B antibodies produced in group O could block viral adhesion to cells and explain a lower risk of infection.

## Introduction

Since the end of 2019, SARS-CoV-2 infection has spread rapidly taking on the characteristics of a pandemic, as declared by the World Health Organization (WHO) in March 2020, with a strong negative impact on public health and the global economic system [1] causing approximately 2 years of pandemic more than 760 million confirmed cases and 6.8 million deaths globally [2].

SARS-CoV-2 spreads mainly through respiratory droplets and the respiratory tract attaching to the human angiotensin-converting enzyme 2 (ACE2) receptor and a specific transmembrane serine protease 2 (TMPRSS2) with the spike glycoprotein (S protein) and infects the host cells [3].

The high infectivity and contagiousness of the virus together with a high presence of asymptomatic or mildly symptomatic individuals capable of transmitting the infection have led to such a rapid spread of the disease [4].

COVID-19 is a disease with a wide spectrum of clinical features: respiratory, neurological, and gastrointestinal symptoms are the most commonly reported symptoms in SARS-CoV-2-infected individuals followed by those involving organs such as skin and subcutaneous tissue, kidneys, cardiovascular system, liver, and eyes found in a limited number of individuals [5].

In many studies, older age and male gender have been reported as important predictors of SARS-CoV-2 infection [6, 7]; ethnicity, obesity, dementia, renal, and cerebrovascular disease have also been identified as important risk factors for SARS-CoV-2 infection along with hormonal, environmental, social, and genetic factors [8–10].

Among other risk factors, blood groups were recognized to confer differential susceptibility to bacteria, viruses, and parasites as certain blood group antigens act as receptors [11–13].

During the previous SARS-CoV-1 outbreak in 2003, Cheng et al. had demonstrated this association, showing a lower risk of infection for blood group O individuals [14].

Soon after the beginning of the pandemic by SARS-CoV-2, a study from Wuhan, China, reported a higher risk of infection for people of blood group A and a lower risk for people of blood group O [15].

Since then, the association between ABO blood groups, which are characterized by the presence of oligosaccharides that determine a specific antigen on the surface of red blood cells (RBCs) and a large number of cell types, and by three antibodies (anti-A, anti-B, anti-A,B) in blood plasma [16], and the risk of SARS-CoV-2 infection has been evaluated in several studies in other parts of the world with results showing discrepancies [17–21].

Although the reverse transcription-polymerase chain reaction (RT-PCR) is the gold standard method for the detection of recent SARS-CoV-2 infection, its sensitivity varies over time as the number of virus particles released by individual changes throughout their infection and does not allow detection of a past viral infection [22].

Serological tests represent a valid biomonitoring system to detect the immunoglobulin M (IgM) and immunoglobulin G (IgG) and to allow an accurate evaluation of previous SARS-CoV-2 infection and circulation of the disease through the community by identifying a population that has been infected by virus and has developed an antibody response [23].

Blood donor cohorts represent a special point of view for the study of subclinical conditions and for describing the prevalence, incidence, and natural course of infectious diseases [24]. Blood donors are a selected population of apparently healthy subjects, aged 18–65 years not affected by active infections or other medical conditions and free of symptoms related to COVID-19, and not to have had any close contact with confirmed cases; therefore, they represent a population of choice to assess the susceptibility to infection.

Therefore, an observational multicentric cross-sectional study aiming at assessing the association between ABO blood groups and SARS-CoV-2 seroprevalence among the blood donors in the Puglia region was performed.

## Materials and methods

The study was carried out on healthy blood donors who donated blood to one of the 22 Transfusion Centers in the Puglia region between September 1, 2020, and February 28, 2021, and whose samples were processed by the three Regional Biological Qualification Centres (BQC) of blood and blood hemocomponents of Bari, Lecce, and Foggia.

The following Transfusion Centres pertain to the BQC of the North Vast Area of Puglia region: Immunohematology and Transfusion Medicine Services (ITMS) of University Hospital OORR Foggia; IRCCS Foundation “Casa Sollievo della Sofferenza,” San Giovanni Rotondo, Foggia; and “Mons. R. Dimiccoli” Hospital, Barletta; Transfusion Centers of “L. Bonomo” Hospital, Andria; “T. Masselli-Mascia” Hospital, San Severo, Foggia; “G. Tatarella” Hospital Cerignola, Foggia; and “San Camillo De Lellis” Hospital, Manfredonia, Foggia.

The following Transfusion Centres in the North and South Areas of the city of Bari belong to the BQC of the Central Vast Area of Puglia region: ITMS of “San Paolo” Hospital, Bari; University Hospital Consortium Corporation Polyclinic of Bari, Bari; “Di Venere” Hospital, Bari; “F. Miulli” General Regional Hospital, Acquaviva delle Fonti, Bari; Transfusion Centers of “San Giacomo” Hospital, Monopoli, Bari; “Don Tonino Bello” Hospital, Molfetta, Bari; and “Fabio Perinei” Murgia Hospital, Altamura, Bari.

Finally, the following Transfusion Centres: ITMS of “Vito Fazzi” Hospital, Lecce; “A. Perrino” Hospital, Brindisi; “S. G. Moscati” Hospital, Taranto; “Sacro Cuore di Gesù” Hospital, Gallipoli, Lecce; “Card. G. Panico” Hospital, Tricase, Lecce; Transfusion Centers of “F. Ferrari” Hospital, Casarano, Lecce; “San Giuseppe da Copertino” Hospital, Copertino, Lecce; and “S. Caterina Novella” Hospital, Galatina, Lecce belong to the BQC of South Vast Area of Puglia region.

Data on ABO and Rh blood groups of all blood donors and their demographic characteristics (age, sex, and time period) were obtained from the Blood Bank Information System.

All patients were asymptomatic at the time of admission to blood donation.

Testing of RBCs for detection of ABO/RhD blood typing was performed by standard automated gel column agglutination method with Ortho BioVue System ABO/D/CDE (Ortho-Clinical Diagnostics, Inc. 1001 US Highway 202 Raritan, NJ 08869 USA).

The Abbott SARS-CoV-2 IgG test assay was used for antibody detection. The assay is a chemiluminescent microparticle immunoassay (CMIA) used for the qualitative detection of anti-nucleocapsid protein IgG antibodies to SARS-CoV-2 in serum samples. The assays were performed on the Alinity ii system (Abbott) and the Abbott Architect i1000 instrument following the manufacturer’s instructions. Samples with a signal-to-cutoff (S/CO) ratio ≥ 1.4 were considered positive.

The comparison of seroprevalence among blood groups and the association between the recorded variables and seroprevalence were evaluated.

The study was notified to the Ethics Committee of Lecce Local Health Unit (LHU), with Prot. n.117023 of 22 July 2021.

### Statistical analysis

Characteristics of individuals were reported as the mean and the standard deviation (SD) for numerical variables and as the absolute and percentage frequencies for categorical variables. The association between the blood groups and seroprevalence was assessed with an individual participant data meta-analysis (IPD MA). The IPD MA was carried out by fitting unadjusted and confounder-adjusted log-binomial mixed models. A random intercept term was included in mixed models to account for correlations among individuals in the same center, while random slope terms for differential effects of blood groups across centers were assessed for inclusion based on AIC and BIC values. Variables that were adjusted for included age at blood sample collection (years), sex (male, female), Rh (positive, negative), and time period (days from the onset of the pandemic). Results were expressed as the prevalence ratio (PR) with 95% confidence intervals (CI), for blood groups A, AB, and B compared to blood group O (reference category). Effect modification was assessed by the inclusion of interaction terms in the adjusted model. Interaction terms for each independent variable were tested globally with a likelihood ratio test and individually with Wald tests on interaction coefficients. Stratified results for effect modifiers were calculated using linear combinations of model coefficients. Analyses were carried out with R 3.6.3 statistical software (The R Foundation for Statistical Computing, Wien) at a significance level equal to *p* < 0.05*.*

## Results

Data on 36,006 individuals were screened, and 297 records were excluded due to missing information (age, Rh). A total of 35,709 blood donors from 22 centers were included in the study. The mean age of the donors was 43.9 years with 73.1% being males (Table 1) with an overall seroprevalence of 6.8% (Table 2).

**Table 1:** Descriptive characteristics of included individuals

Characteristics of subjects	Group 0 (*n* = 16,720)	Group A (*n* = 12,147)	Group AB (*n* = 1602)	Group B (*n* = 5240)	*p*-value
Age, years (mean ± SD)	43.8 ± 12.1	44.0 ± 11.9	44.3 ± 11.7	44.2 ± 11.9	0.2164
Sex, male (*n* %)	12,184 (72.9%)	8954 (73.7%)	1176 (73.4%)	3788 (72.3%)	0.2037
Rh, positive (*n* %)	14,605 (87.4%)	10,853 (89.3%)	1428 (89.1%)	4704 (89.8%)	0.0000
Time period*, days (mean ± SD)	89.2 ± 47.5	89.6 ± 47.5	88.5 ± 47.1	88.2 ± 47.6	0.3184
Seroprevalence (*n* %)	1042 (6.2%)	911 (7.5%)	120 (7.5%)	343 (6.5%)	-

*SD* standard deviation, *days since 22/2/2020

**Table 2:** Seroprevalence in each included center

Center	SARS-CoV-2 negative		SARS-CoV-2 positive	
	*n*	%	*n*	%
North vast area				
“L. Bonomo” Hospital, Andria	705	92.9%	54	7.1%
“Mons. R. Dimiccoli” Hospital, Barletta	1900	93.0%	143	7.0%
“G. Tatarella” Hospital Cerignola, Foggia	372	88.6%	48	11.4%
“San Camillo De Lellis” Hospital, Manfredonia, Foggia	276	98.9%	3	1.1%
University Hospital “OORR,” Foggia	3956	91.2%	383	8.8%
IRCCS Foundation “Casa Sollievo della Sofferenza,” San Giovanni Rotondo, Foggia	3543	91.5%	328	8.5%
“T. Masselli-Mascia” Hospital, San Severo, Foggia	763	87.5%	109	12.5%
Central vast area				
“F. Miulli” General Regional Hospital, Acquaviva delle Fonti, Bari	361	93.3%	26	6.7%
“Fabio Perinei” Murgia Hospital, Altamura, Bari	280	80.0%	70	20.0%
“Di Venere” Hospital, Bari	1052	94.4%	62	5.6%
“San Paolo” Hospital, Bari	1535	91.1%	150	8.9%
“Don Tonino Bello” Hospital, Molfetta, Bari	1333	96.5%	49	3.5%
“San Giacomo” Hospital, Monopoli, Bari	1164	98.1%	23	1.9%
University Hospital Consortium Corporation Polyclinic of Bari, Bari	2075	84.0%	396	16.0%
South vast area				
“A. Perrino” Hospital, Brindisi	3346	94.3%	204	5.7%
“F. Ferrari” Hospital, Casarano, Lecce	204	98.1%	4	1.9%
“San Giuseppe da Copertino” Hospital, Copertino, Lecce	261	96.0%	11	4.0%
“Vito Fazzi” Hospital, Lecce	2343	96.1%	95	3.9%
“S. Caterina Novella” Hospital, Galatina, Lecce	1722	99.2%	14	0.8%
“Sacro Cuore di Gesù” Hospital, Gallipoli, Lecce	1016	97.7%	24	2.3%
“S.G. Moscati” Hospital, Taranto	4273	95.3%	209	4.7%
“Card. G. Panico” Hospital, Tricase, Lecce	813	98.7%	11	1.3%

Among the 8576 blood donors belonging to the 7 centers of the Central Vast Area of Puglia region, 9.0% were positive. A similar seroprevalence was observed in Blood Banks of the North Vast Area (8.5%) among the 12,583 referring blood donors. The lowest overall seroprevalence of 3.9% was observed in South Vast Area among 14,550 blood donors (Table 2).

The most frequent ABO phenotype in included population was blood type O (46.8%), followed by blood type A (34.0%) and B (14.7%), and finally, the least common was group AB with 4.5% of individuals belonging to it.

Rh-positive individuals accounted for 88.4% of the blood donors.

Among the total of subjects reactive for anti-SARS-CoV-2 IgG (2416 blood donors), 43.1% was of blood type O; 37.7% of blood type A; 14.2% of blood type B, and finally, 5% of blood type AB.

The seroprevalence of phenotype A and AB was the highest (7.5%), followed by phenotype B (6.5%) and phenotype 0 with 6.2% of positive individuals (Table 1).

We decided against the inclusion of random slope terms in mixed models, as both the AIC and BIC values for the models without random slopes were smaller than the ones from the models with random slopes.

According to the adjusted analysis, there was an increase in seroprevalence in blood groups A and AB, compared to group O, whereas no difference was observed between groups B and 0. The increase in seroprevalence was very similar in groups A and AB, being equal to + 22.4% (PR = 1.224, 95% CI = 1.128–1.330, *p* < 0.0001) and + 25.2% (PR = 1.252, 95% CI = 1.051–1.490, *p* = 0.0116), respectively (Table 3).

**Table 3:** Comparison of seroprevalence among blood groups

	Unadjusted			Adjusted*		
Group	PR	95% CI	*p*-value	PR	95% CI	*p*-value
0	-	-	-	-	-	-
A	1.235	1.136–1.345	0.0000	1.224	1.128–1.330	0.0000
AB	1.236	1.033–1.479	0.0204	1.252	1.051–1.490	0.0116
B	1.040	0.927–1.169	0.5000	1.052	0.939–1.178	0.3823

*PR* prevalence ratio, *CI* confidence interval; *adjusted for age, sex, Rh, and time period

An increase in seroprevalence in males was observed compared to the female population (PR = 1.105, 95% CI = 1.014–1.206, *p* = 0.0234), whereas no difference was found between positive and negative Rh factor (PR = 1.076, 95% CI = 0.954–1.212, *p* = 0.2318) (Table 4).

**Table 4:** Association between the recorded variables and seroprevalence, according to the adjusted analysis

Variable	Multivariable model		
	PR	95% CI	*p*-value
Blood group	-	-	-
A vs 0	1.224	1.128–1.330	0.0000
AB vs 0	1.252	1.051–1.490	0.0116
B vs 0	1.052	0.939–1.178	0.3823
Age	-	-	-
10-year increment	0.942	0.913–0.971	0.0001
Sex	-	-	-
Male vs female	1.105	1.014–1.206	0.0234
Rh	-	-	-
Pos. vs Neg	1.076	0.954–1.212	0.2318
Time period *	-	-	-
1-month increment	1.564	1.520–1.608	0.0000

*PR* prevalence ratio, *CI* confidence interval; *days since 22/2/2020

A possible effect modification was observed after stratifying for sex (*p* = 0.0515), whereas no modification was observed for age (*p* = 0.3797), Rh (*p* = 0.8367), and time period (*p* = 0.7784) (Table 5). In the stratified analysis, blood group A had a higher seroprevalence for both males (PR = 1.236, 95% CI = 1.123–1.359, *p* < 0.0001) and females (PR = 1.198, 95% CI = 1.017–1.412, *p* = 0.0308), whereas blood group AB had a higher seroprevalence in males (PR = 1.374, 95% CI = 1.135–1.663, *p* = 0.0011) but not in females (PR = 0.879, 95% CI = 0.577–1.339, *p* = 0.5473). Blood group B was not associated with seroprevalence in both strata (*p* = 0.0698 for males and *p* = 0.1692 for females) (Table 6).

**Table 5:** Assessment of effect modification

Characteristics of subjects	Interaction term (*p*-value)			
	Group A	Group AB	Group B	Global test
Age	0.7492	0.1238	0.3104	0.3797
Sex	0.7512	0.0583	0.0390	0.0515
Rh	0.3981	0.7219	0.5720	0.8367
Time period	0.4161	0.5385	0.4401	0.7784

**Table 6:** Comparison of seroprevalence among blood groups, stratified by sex

Group	Males			Females		
	PR	95% CI	*p*-value	PR	95% CI	*p*-value
0	-	-	-	-	-	-
A	1.236	1.123–1.359	0.0000	1.198	1.017–1.412	0.0308
AB	1.374	1.135–1.663	0.0011	0.879	0.577–1.339	0.5473
B	1.126	0.990–1.281	0.0698	0.843	0.661–1.075	0.1692

*PR* prevalence ratio, *CI* confidence interval; the model is adjusted for age, Rh, and time period

## Discussion

Numerous risk factors responsible for an increased vulnerability to SARS-CoV-2 infection were identified [25]. Among others, the role of the ABO blood type and Rhesus status have been suggested as factors for variable predisposition to COVID-19 [26, 27].

Studies on the relationship between ABO and Rh blood types with SARS-CoV-2 infection are not consistent in their findings, and there is no evidence in the literature on the underlying mechanisms.

Our study suggests a potential influence of ABO type on SARS-CoV-2 susceptibility in a large regional cohort of blood donors that ensures a high degree of homogeneity of results.

An increase in seroprevalence in blood groups A and AB, compared to group O, was found. Similarly, previous studies have found that the ABO blood system is linked to significantly different susceptibility to other viral infections related to the expression of ABH antigens and the correspondent absence or presence of anti-A and anti-B antibodies which may provide a different defense against infections [28, 29].

Several authors have shown that blood group O is correlated to decreased susceptibility to SARS-CoV-2 infection [29–33], and individuals with blood group A were at higher risk [17, 30, 31, 34], while discordant results concern the higher risk for infection in subjects with blood group AB.

One systematic review has reported indication of blood type AB linked to a lower risk of COVID-19 infection [35]. Other authors have, however, observed a lower susceptibility to infection in subjects with group O and AB [36, 37] or a greater risk of infection in those of group A and lower in subjects with blood group AB and O [38].

In a recent review that aimed to summarize the literature regarding the association between ABO blood types and COVID-19, in most studies (21 of 38 studies), the association between blood type O and lower susceptibility to infection and between blood type A and increased risk of acquiring the infection (in 18 of 38 studies) was found, whereas blood type AB was associated with a higher risk in only 10 out of 38 studies [27].

In particular, in a study conducted among a sample of SARS-CoV-2-positive individuals in Bahrain, blood group AB was associated with a lower risk of infection [39]. In this study, the control group consisted of blood or platelet donors, representative of the general population.

Discrepant results mainly related to blood group AB may be due to the fact that group AB is the rarest ABO type with a prevalence of 5% [17, 31]. Conflicting results may also reflect the small sample size or their design.

Another source of conflicting results could be due to the mixing of cohorts, as in case–control studies in which cases and controls belong to different populations. Also, because ABO-type frequencies differ in different populations and ethnic groups, the results of an association study in one region are different in other ethnic groups. Studies with small sample sizes are unlikely to produce meaningful evidence, how, while studies on large cohorts with several thousand subjects from a homogeneous population can help to clarify the association between ABO blood type and COVID-19 infection.

Several authors have highlighted the usefulness of blood donors as an accessible and representative population for performing epidemiological surveys in emerging infectious diseases [40, 41].

In our study, we considered the distribution of blood groups among the subjects reactive for anti-nucleocapsid protein SARS-CoV-2 IgG antibodies infection induced in 35,709 healthy blood donors who donated blood to one of the 22 Transfusion Centers in the Puglia region between September 1, 2020, and February 28, 2021. The assay for the detection of N antibodies permits to differentiate infection- and vaccination-induced seropositivity [42].

Overall, 46.8% of donors were blood group O, followed by 34% of blood group A, 14.7% of blood group B, and 4.5% of blood group AB; this distribution reflects the distribution of blood groups in the general population of Southern Italy [43]. Therefore, blood donors can be used to monitor the susceptibility and evolution of SARS-CoV-2 infection [44, 45].

Blood groups are determined by specific complex carbohydrates present on the extracellular surface of RBC; N-acetyl-d-galactosamine for the A antigen, and d-galactose for the B antigen. Both are present on the H antigen, which defines the blood type O. Although ABH antigens are considered RBC antigens, they are expressed in a wide variety of human tissues, are present on the surface of most human epithelial and endothelial cells, and may act as ligands for pathogens [46].

Blood groups A and AB are determined by the presence of A antigen; therefore, the higher risk of SARS-CoV-2 infection in people with blood type A and AB could be due to the role of antigen A in the binding of SARS-CoV-2 to its ACE2 receptor [47]. Moreover, the association between the ABO system and the frequency of certain infectious diseases can also be affected by the presence of iso-hemagglutinins [12].

Our data suggest a lower seroprevalence of the infection in group O and B subjects; therefore, the anti-A iso-hemagglutinins present in group O and B subjects and absent in individuals of group A and AB could have a protective role against SARS-CoV-2 infection, as they are capable of inhibiting the viral entry into the host cell as already demonstrated for SARS-CoV-1 infection [48, 49].

The underlying mechanisms may include ABH antigens as co-receptors for pathogens and natural antibodies as inhibitors of pathogen-host binding [50]. The higher risk of infection in people with blood type A could be due to the role of substance A in the binding of SARS-CoV-2 to its receptor, with a mechanism similar to that of surface heparin sulfates and sialic acid [51]. Natural isoagglutinins may play a protective role against infection and it is conceivable that higher titers of isoagglutinins may confer greater protection than subjects with lower titers, as demonstrated by Guillon P. et al. during the SARS-CoV-1 epidemic [48].

Some authors have also hypothesized a role of the secretory state on different susceptibility of ABO blood types to the SARS-CoV-2 infection [52, 53], although, in a recent large cohort study of blood donors, no interaction between secretor status and ABO blood group COVID susceptibility was found [54].

Future studies that take into consideration both the secretory state and the agglutinins with reference also to their titers could clarify their potential pathogenic role in the infection.

Our results are supported by a consistent population of analyzed subjects; therefore, they give strength to the hypothesis that suggests the ABO blood type is a useful biomarker to predict the risk of infection and to estimate the dynamics of virus transmission.

Additionally, our results show an increase in seroprevalence in males compared to the female population. In numerous studies, greater susceptibility to SARS-CoV-2 of males compared to females has been observed and the male sex is considered a risk factor for both morbidity and mortality in COVID-19 [55–57].

The discrepancy could be explained by a combination of lifestyle/behavioral, biological, and genetic factors.

Men are more likely to engage in poor health behaviors such as smoking and alcohol consumption and to leave the house and frequently crowded areas [58].

Furthermore, the difference in immunological defense and immune responses, genetics, and hormones represent major factors sex related to COVID-19 susceptibility and severity.

Chromosomal differences, together with differential expression of the ACE2 and TMPRSS2 genes, could explain the difference in both the susceptibility and outcome of COVID-19 disease, although the mechanisms are not yet elucidated [58].

In particular, the use of stratified analysis suggested that blood group A had a higher seroprevalence for both sexes, whereas blood group AB had a higher seroprevalence only in males.

The gaps in knowledge regarding the sex differences in COVID-19 especially associated with blood groups should be addressed in future studies.

Our study has some limitations that need to be pointed out. First, in the analyzed cohort, it is possible that the prevalence of SARS-CoV-2 antibodies has been underestimated, as symptomatic subjects were excluded from blood donation.

Furthermore, asymptomatic donors with active infection may have been classified as seronegative if the assay was performed before the onset of a strong humoral immune response against SARS-CoV-2. Despite the high sensitivity and specificity of the ELISA Abbott IgG used [59], some individuals may still have been misclassified. Second, the methodologies used to detect antibodies were qualitative and we did not analyze the difference in changing antibody titers over time. Third, blood donors may represent a population less likely to be exposed to SARS-CoV-2 than the general population. They tend to have better health than the general population, resulting in an effect known as the “healthy donor effect” [60], and recruitment practices and eligibility criteria for blood donations may bias the sample of donors to a lower risk [41]. In particular, due to eligibility criteria in Italy, children aged younger than 18 years and elderly older than 65 years cannot donate blood and were not included in this study.

Last, blood donors as representative of the distribution of the ABO phenotype in the general population can be considered a source of bias because of blood type O being overrepresented among regular donors [27].

Despite these limitations, blood donors can represent an accessible population to detect trends of the epidemic and evaluate any associations between blood group and seroprevalence of the infection.

## Conclusions

A significantly lower prevalence of blood donors with blood type O was found compared to blood types A and AB, whereas no association was observed between the Rh factor and the seroprevalence.

Although the mechanisms that can explain the observed data have not yet been clarified, some hypotheses can be made.

The main one assumes that the A antigen present in people with blood type A and AB may play a role in the binding of SARS-CoV-2 to ACE2 receptors, resulting in an increased risk of SARS-CoV-2 infection.

Furthermore, natural anti-A and anti-B antibodies produced in individuals with blood group O could potentially block viral adhesion to cells, which could explain a lower risk of infection, but more studies are needed to clarify these hypotheses.

## Data Availability

The data that support the findings of this study are not openly available to preserve study participant privacy and are available from the corresponding author upon reasonable request.
